# Building a Bigger Tent – Interprofessional Engagement in Geriatric Curriculum Development

**DOI:** 10.1093/geroni/igaf122.738

**Published:** 2025-12-31

**Authors:** Beth Hogans, Katharina Echt

**Affiliations:** Johns Hopkins School of Medicine, Baltimore, Maryland, United States; U.S. Department of Veterans Affairs, Brookhaven, Georgia, United States

## Abstract

This presentation will provide an overview of curriculum development including needs assessment and collaborative creation of interprofessional learning objectives before describing a national effort building geriatric interprofessional core curriculum for Veterans Administration (VA) health professions trainees. Using modified Delphi processes and multi-level interprofessional feedback, the curriculum development focused on distilling information from the task, needs, and gap assessments and synthesizing a coordinated set of learning objectives to support module development. Following the needs assessment process which included resource appraisal and a series of national stakeholder roundtable meetings, one for each of the geriatrics 5 Ms, we developed and delivered a survey directed to the cohort of associate directors for education and evaluation at the 20 VA Geriatric Research Education and Clinical Centers of Excellence (GRECCs) nationally. The purpose of the survey was to review and finalize the recommendations of the stakeholder meetings, ensuring that topics proposed were relevant to identified education needs across the national program. Subsequently the results of the survey were discussed, and core content topics harmonized across the curriculum. Once the curriculum topics were finalized, interprofessional curriculum team members were assigned to draft learning macro and micro-objectives designed to describe the specifics of the course content. Draft learning objectives were presented to the curriculum team as a whole and underwent multiple rounds of editing for alignment of purpose, clarity and concision. National education experts provided additional peer-review feedback and learning objectives were reviewed by diverse VA education stakeholders. Final learning objectives were utilized to shape module development.

